# Quantitative characterization of breast lesions and normal fibroglandular tissue using compartmentalized diffusion-weighted model: comparison of intravoxel incoherent motion and restriction spectrum imaging

**DOI:** 10.1186/s13058-024-01828-3

**Published:** 2024-04-24

**Authors:** Litong He, Yanjin Qin, Qilan Hu, Zhiqiang Liu, Yunfei Zhang, Tao Ai

**Affiliations:** 1 Department of Radiology, Tongji Hospital, Tongji Medical College, Huazhong University of Science and Technology, NO. 1095 Jiefang Avenue, Qiaokou District, Wuhan, 430030 China; 2 Department of Radiology, The First Affiliated Hospital, Sun Yat-sen University, 58th the Second Zhongshan Road, Guangzhou, 510080 China; 3 MR Collaboration, Central Research Institute, United Imaging Healthcare, Shanghai, China

**Keywords:** Diffusion-weighted imaging, Intravoxel incoherent motion, Restriction spectrum imaging, Breast lesion

## Abstract

**Background:**

To compare the compartmentalized diffusion-weighted models, intravoxel incoherent motion (IVIM) and restriction spectrum imaging (RSI), in characterizing breast lesions and normal fibroglandular tissue.

**Methods:**

This prospective study enrolled 152 patients with 157 histopathologically verified breast lesions (41 benign and 116 malignant). All patients underwent a full-protocol preoperative breast MRI, including a multi-b-value DWI sequence. The diffusion parameters derived from the mono-exponential model (ADC), IVIM model (Dt, Dp, f), and RSI model (C_1_, C_2_, C_3_, C_1_C_2_, F_1_, F_2_, F_3_, F_1_F_2_) were quantitatively measured and then compared among malignant lesions, benign lesions and normal fibroglandular tissues using Kruskal-Wallis test. The Mann-Whitney U-test was used for the pairwise comparisons. Diagnostic models were built by logistic regression analysis. The ROC analysis was performed using five-fold cross-validation and the mean AUC values were calculated and compared to evaluate the discriminative ability of each parameter or model.

**Results:**

Almost all quantitative diffusion parameters showed significant differences in distinguishing malignant breast lesions from both benign lesions (other than C_2_) and normal fibroglandular tissue (all parameters) (all *P* < 0.0167). In terms of the comparisons of benign lesions and normal fibroglandular tissues, the parameters derived from IVIM (Dp, f) and RSI (C_1_, C_2_, C_1_C_2_, F_1_, F_2_, F_3_) showed significant differences (all *P* < 0.005). When using individual parameters, RSI-derived parameters-F_1_, C_1_C_2_, and C_2_ values yielded the highest AUCs for the comparisons of malignant vs. benign, malignant vs. normal tissue and benign vs. normal tissue (AUCs = 0.871, 0.982, and 0.863, respectively). Furthermore, the combined diagnostic model (IVIM + RSI) exhibited the highest diagnostic efficacy for the pairwise discriminations (AUCs = 0.893, 0.991, and 0.928, respectively).

**Conclusions:**

Quantitative parameters derived from the three-compartment RSI model have great promise as imaging indicators for the differential diagnosis of breast lesions compared with the bi-exponential IVIM model. Additionally, the combined model of IVIM and RSI achieves superior diagnostic performance in characterizing breast lesions.

## Introduction

Diffusion-weighted magnetic resonance imaging (DW-MRI) is a non-invasive imaging technique that has gained widespread use in clinical and research settings [1, 2]. Conventional DWI provides information about the random Brownian motion of water particles in microscopic biological tissues by calculating the quantitative apparent diffusion coefficient (ADC) through the mono-exponential diffusion model and is thought to have the ability to probe tumor cellularity [3]. Although ADC value has shown promising clinical utility as an imaging biomarker for characterizing breast tumors [4, 5], there still exist noticeable limitations, such as the mixture of water molecules diffusion signal from intracellular and extracellular, the significant overlap of ADC values for benign and malignant lesions, and the geometric distortion, which is a common limitation shared by various DWI techniques [6, 7].

Compartmentalized diffusion models, which separate each voxel into multiple compartments based on tissue microstructural or diffusion properties, can partially address these limitations of conventional DWI and provide additional information for assessing water molecule diffusion in the tissue [8]. One of the compartmentalized models, Intravoxel Incoherent Motion (IVIM), which divided the diffusion signal into two distinct components, microcirculation perfusion and tissue diffusivity, is hypothesized to characterize both tissue cellularity and micro-vascularity by measuring the tissue diffusion coefficient (Dt) and perfusion-related parameters of pseudo-diffusion coefficient (Dp) and perfusion fraction (f) [9]. Previous studies have investigated the potential utility of the bi-exponential IVIM model in the identification of breast lesions [10–13]. Another compartmentalized model is Restriction Spectrum Imaging (RSI). In breast-specific RSI, the diffusion signal is modelled as a mixture of three compartments corresponding to intracellular restricted, extracellular hindered, and free water pools, which can be quantified through the signal contributions parameters C_1_, C_2_, C_3_ and signal fractions parameters F_1_, F_2_, F_3_ respectively [14]. The individual weights of the three components can be decomposed, and the slowly intracellular restricted water component can theoretically be isolated by applying a generalized linear estimation technique and extended b-values [15, 16]. In this context, the underlying tissue characteristics, such as cellularity, nuclear volume fraction, and microstructure, can be quantitatively assessed with RSI DWI [17]. It has been proven that RSI can improve the conspicuity of highly cellular lesions, which may be helpful in breast cancer screening [18]. Other studies have demonstrated its clinical applications in prostate and breast [19–23]. Although these studies have shown that the RSI model has relatively better diagnostic performance for discriminating breast lesions, the comparison and integration of RSI and IVIM models would likely lead to a new perspective beyond what each individual parameter or model can provide by taking full advantage of the diffusion data from low to high b-values.

Therefore, this study aims to compare the compartmentalized diffusion-weighted models, intravoxel incoherent motion (IVIM) and restriction spectrum imaging (RSI), in characterizing breast lesions and normal fibroglandular tissue, and then to identify optimal imaging biomarkers or models to facilitate the clinical diagnosis of breast cancer.

## Materials and methods

### Patient population

The Institutional Review Board (IRB) of our hospital approved this prospective study (TJ-IRB20230948), and informed consent was obtained from each subject before the breast MRI examination. Between July 2022 and April 2023, 248 consecutive patients who underwent the routine breast MRI examination due to suspicious breast lesions were preliminarily recruited. The inclusion criteria were as follows: (1) mass lesion with a maximal diameter greater than 10 mm; (2) histopathological confirmation by either imaging-guided breast biopsy or surgical resection; (3) no biopsy, surgery, radiotherapy or chemotherapy performed prior to breast MRI. Meanwhile, Ninety-six patients were excluded from this study for the following reasons: 38 had lesions with non-mass morphological characteristics; 8 had breast implants; 26 had mass lesions with a diameter smaller than 10 mm; 24 had poor image quality due to significant motion artifacts or insufficient fat saturation on RSI-DWI images. In addition, five patients had synchronous malignancy and benign lesions [ipsilateral (*n* = 2) and contralateral (*n* = 3)]. Finally, 152 patients (median age, 49 years; age range, 19–76 years) with 157 breast lesions (41 benign and 116 malignant) were included in this study. The clinical and pathological data was retrieved from the electronic medical records of the Hospital Information System (HIS) for each subject.

### MRI acquisition

Each subject underwent a bilateral breast MRI examination using a 3.0T MR scanner (uMR 790, United Imaging Healthcare, China) equipped with a 10-channel breast surface coil in the prone position. The principle imaging protocol included a T2-weighted fast spin-echo (FSE) sequence, an axial multi-b-value DWI sequence with reduced field of view (FOV) single-shot echo-planar imaging (ss-EPI), specifically utilizing Microview DWI, and a T1-weighted gradient-echo DCE-MRI sequence. The detailed sequence parameters are provided in Table 1.

**Table 1:** Acquisition parameters for T2WI, DCE-MRI, and DWI sequences

Parameters	T2WI	DCE-MRI	DWI
Repetition time (msec)	2500	3.59	2000
Echo time (msec)	92	1.51	61.5
Flip angle (°)	90	10	90
Field of view (mm^2^)	320 × 320	300 × 300	320 × 160
Matrix	320 × 272	176 × 176	192 × 192
Slice thickness (mm)	4	2	5
Pixel bandwidth (Hz/Px)	220	660	1500
Temporal resolution (sec/phase)	N/A	11.55	N/A
b-values (sec/mm^2^)	N/A	N/A	0, 50, 100, 250, 500, 750, 1000, 1500, 2000
Gradient directions	N/A	N/A	3
Phase-encoding (PE) direction	Right-Left (R/L)	Right-Left (R/L)	Anterior-Posterior (A/P)
Acquisition time (sec)	145	462	330

Note: T2WI, T2-weighted imaging; DCE-MRI, dynamic contrast enhanced-magnetic resonance imaging; DWI, diffusion weighted imaging; N/A, not available

### Image analysis

Breast MRI reports were reviewed to record the morphological characteristics of each breast lesion. Before the lesion segmentation, DWI data was pre-processed by correction for geometric distortion, eddy current artifacts, and gradient nonlinearities [24]. Specifically, geometric distortion correction was performed utilizing a simple gradient field model, supplemented by imaging information derived from the geometry of gradient coils, following the methods proposed by Serge Langlois [25]. Additionally, we employed gradient field spherical harmonic expansion-based deconvolutional methods to mitigate negative impacts caused by non-linear magnetic field gradients, as per a previously reported strategy [26]. Furthermore, eddy current correction was executed by characterizing the field inhomogeneities from a field map and subsequently unwrapping the images to achieve accurate alignment with conventionally collected images [27].

First of all, the regions of interest (ROI) of the whole lesion volume were manually delineated along the outer contour of the lesion on transversal DWI images at b = 750 s/mm^2^ by two independent radiologists (Y.J.Q. and T.A., with 3 and 11 years of experience in breast imaging interpretation, respectively) using ITKsnap software (version 3.8.0; http://www.itksnap.org), under the guidance of all available imaging data in conventional MRI protocol (including T2 images and DCE-MRI) to accurately locating the lesions and confirming the boundaries. Meanwhile, another volumetric ROI with a diameter of 10 mm was manually placed on the normal fibroglandular tissues of the contralateral breast. Subsequently, both ROIs were automatically propagated to the ADC maps, IVIM parametric maps, and RSI parametric maps for calculating diffusion parameters using in-house software on Matlab R2018b (MathWorks Inc., Natick, MA, USA).

The ADC value with the mono-exponential model was calculated based on the following equation [28]:

$${S_b}/{S_0} = {e^{ - b \cdot ADC}}$$

where S_0_ represents the baseline signal intensity without diffusion weighting, and S_b_ is the signal intensity with b-values of 750 s/mm^2^.

IVIM-derived parameters were obtained using the bi-exponential diffusion model:

$${S_b}/{S_0} = \left( {1 - f} \right) \cdot {e^{ - b \cdot Dt}} + f \cdot {e^{ - b \cdot \left( {Dt + Dp} \right)}}$$

where S_b_ represents the diffusion-weighted signal intensity obtained from the given b-value (0, 50, 100, 250, 500, 750, 1000 s/mm^2^). Dt, true diffusion coefficient (unit: mm^2^/s), reflects the component of slow water molecular movement in the extravascular tissue; Dp, pseudo-diffusion coefficient (unit: mm^2^/s), is related to fast water motion in the capillaries; and f, perfusion fraction (0 ≤ f ≤ 1), corresponds to the volume fraction of the microvasculature in a voxel.

Quantitative parameters of RSI-DWI were obtained using the following three-exponential model as utilized by Andreassen et al. previously [23]:

$${S_b} = {C_1} \cdot {e^{ - b \cdot AD{C_1}}} + {C_2} \cdot {e^{ - b \cdot AD{C_2}}} + {C_3} \cdot {e^{ - b \cdot AD{C_3}}}$$

where S_b_ is the signal intensity at a specific b-value, and C_i_ (i = 1, 2, and 3) denotes the signal contribution of each particular component of the three-exponential breast model separately. More precisely, in the matter of breast lesions, the parameter C_1_ describes the most restricted diffusion in intracellular space, which corresponds to excessive cellularity in tumor; C_2_ represents hindered diffusion primarily from extracellular regions or fibroglandular tissue; C_3_ is associated with the least restricted diffusion derived from free water diffusion or blood flow [17, 18]. The ADC_1,_ ADC_2_, and ADC_3_ were established to 0 mm^2^/sec, 1.4 × 10^− 3^ mm^2^/sec, and 10.2 × 10^− 3^ mm^2^/sec, which have been demonstrated to be the best fit of breast tissue and lesion [22, 23].

By normalizing the voxel signal at b = 0 s/mm^2^, signal fractions could generate correspondingly:

$${S_b}/{S_0} = {F_1} \cdot {e^{ - b \cdot AD{C_1}}} + {F_2} \cdot {e^{ - b \cdot AD{C_2}}} + {F_3} \cdot {e^{ - b \cdot AD{C_3}}}$$

where the sum of F_1_, F_2_, and F_3_ is 1. On account of eliminating the effect of proton density- and T2-weighted, the signal fraction directly correlates with diffusion component effects [23].

The quantitative signal contributions C_1_, C_2_, C_3_ and the signal fractions F_1_, F_2_, F_3_ could be directly calculated from the models. C_1_C_2_ and F_1_F_2_, the corresponding product of C_1_, C_2_ and F_1_, F_2_, were shown to have the capacity to distinguish breast cancers from the normal tissues and benign lesions and were also embraced in this research [17, 22].

In the present study, the mono-exponential model was fitted using the least-squares fit for linear fitting and the intravoxel incoherent motion (IVIM) and restriction spectrum imaging (RSI) models followed the Levenberg–Marquardt fit for nonlinear fitting, which were commonly used fitting algorithms as described in previous studies [29].

### Statistical analysis

In this study, statistical analysis was conducted using SPSS 27.0 (IBM, Armonk, NY, USA) and GraphPad Prism 9.0 (GraphPad Inc., San Diego, CA, USA). The Dice similarity coefficient (DSC), recall, and precision were calculated to evaluate the geometric overlap of the regions of interest (ROIs) drawn by the two readers. Additionally, we have utilized the intraclass correlation coefficient (ICC) to assess the consistency of each diffusion parameter measured by the two readers. The results were interpreted based on the following criteria: excellent agreement (0.81-1.00), good agreement (0.61–0.80), moderate agreement (0.41–0.60), fair agreement (0.21–0.40), and slight agreement (0.01–0.20). The Kolmogorov-Smirnov normality test was performed for all quantitative diffusion parameters, and the Kruskal-Wallis rank sum test was used to compare the individual parameters derived from the mono-exponential, IVIM, and RSI models among the three different types of breast tissues. Subsequently, the Mann-Whitney U-test was conducted to detect the difference between malignant vs. benign lesions, malignant lesions vs. normal fibroglandular tissue, and benign lesions vs. normal fibroglandular tissue, and the statistically significant difference was set at a *P*-value less than 0.0167 to mitigate the risk of false positives due to multiple comparisons.

The study established IVIM model (Dt + Dp + f), RSI model (C_1_ + C_2_ + C_3_ + C_1_C_2_ + F_1_ + F_2_ + F_3_ + F_1_F_2_), and hybrid model (IVIM + RSI) via logistic regression. The performance of each model was evaluated using five-fold stratified cross-validation (CV) to avoid overfitting. Receiver operating characteristic curve (ROC) analysis was applied to evaluate the diagnostic capacities of the individual diffusion parameters and integrated diagnostic models in the discrimination of the different tissue types. The mean area under the curves (AUCs) were compared by the DeLong test (31).

## Results

### Baseline characteristics

A total of 152 patients (median age, 49 years; age range, 19–76 years) with 157 breast lesions were included. Of the 157 breast lesions, there were 41 benign (21 fibroadenomas, 10 adenoses, 5 intraductal papillomas, 3 benign phyllodes tumors, 1 granulomatous mastitis, and 1 usual ductal hyperplasia) and 116 malignant (96 invasive ductal carcinomas, 12 ductal carcinomas in situ, 4 invasive micropapillary carcinomas, 2 invasive lobular carcinomas, 1 invasive cribriform carcinoma, and 1 carcinomas with medullary feature). The detailed characteristics of lesions were summarized in Table 2.

**Table 2:** Baseline characteristics and histopathologic results of Lesions

	Benign lesions (*N* = 41)		Malignant lesions (*N* = 116)		P value
Mean age (years)	42.1 ± 11.6 (19–64)		50.7 ± 11.8 (26–76)		<0.001*
Menstruation state					<0.001*
Premenopausal	26 (63%)		38 (33%)		
Postmenopausal	15 (37%)		78 (67%)		
Side					0.640
Left	24 (59%)		63 (54%)		
Right	17 (41%)		53 (46%)		
Mean diameter (mm)	22.1 ± 17.1 (11.0-100.0)		26.0 ± 9.1 (10.0–60.0)		<0.001*
Histologic result (no.)	Fibroadenoma	21 (51%)	Invasive ductal carcinoma	96 (83%)	
	Adenoses	10 (24%)	Ductal carcinoma in situ	12 (10%)	
	Intraductal papilloma	5 (12%)	Invasive micropapillary carcinoma	4 (3%)	
	Benign phyllodes tumor	3 (7%)	Invasive lobular carcinoma	2 (2%)	
	Granulomatous mastitis	1 (3%)	Invasive cribriform carcinoma	1 (1%)	
	Usual ductal hyperplasia	1 (3%)	Carcinomas with medullary feature	1 (1%)	
Shape					<0.001*
Round	23 (56%)		10 (8%)		
Oval	12 (29%)		3 (3%)		
Irregular	6 (15%)		103 (89%)		
Enhancement					<0.001*
Homogeneous	30 (73%)		22 (19%)		
Heterogeneous	8 (20%)		90 (78%)		
Rim	3 (7%)		4 (3%)		

Note: Data presents mean ± SD or the proportion of lesions, with the range or percentage in parentheses. *P value less than 0.05

### Assessment of inter-observer agreement

The assessment of inter-observer agreement of the segmentation of ROIs and mono-exponential, IVIM, and RSI-derived parameters was conducted in this study. For delineation of ROIs, the mean DSC was 0.885 ± 0.099, while the mean recall and precision were 0.948 ± 0.065 and 0.846 ± 0.152, respectively. For measurement of parameters, the results showed that the agreement between the two readers was excellent, with ICCs greater than 0.8 (ranging from 0.863 to 0.993). Subsequently, statistical analysis was performed using the measurement result from one of the two readers. The detailed results are provided in Table 3.

**Table 3:** Interobserver agreement for measurements of diffusion parameters

	ICC	95% Confidence Interval	P Value
Mono-ADC	0.947	0.933–0.958	<0.001
IVIM-Dt	0.953	0.939–0.963	<0.001
IVIM-Dp	0.979	0.974–0.983	<0.001
IVIM-f	0.894	0.868–0.915	<0.001
RSI-C_1_	0.987	0.984–0.990	<0.001
RSI-C_2_	0.984	0.979–0.987	<0.001
RSI-C_3_	0.978	0.972–0.982	<0.001
RSI-C_1_C_2_	0.993	0.991–0.995	<0.001
RSI-F_1_	0.863	0.822–0.893	<0.001
RSI-F_2_	0.866	0.828–0.895	<0.001
RSI-F_3_	0.961	0.952–0.969	<0.001
RSI-F_1_F_2_	0.933	0.913–0.948	<0.001

Note: ICC, intraclass correlation coefficients

### Comparative analysis of diffusion parameters among malignant tumors, benign lesions, and normal fibroglandular tissues

**Fig. 1:**
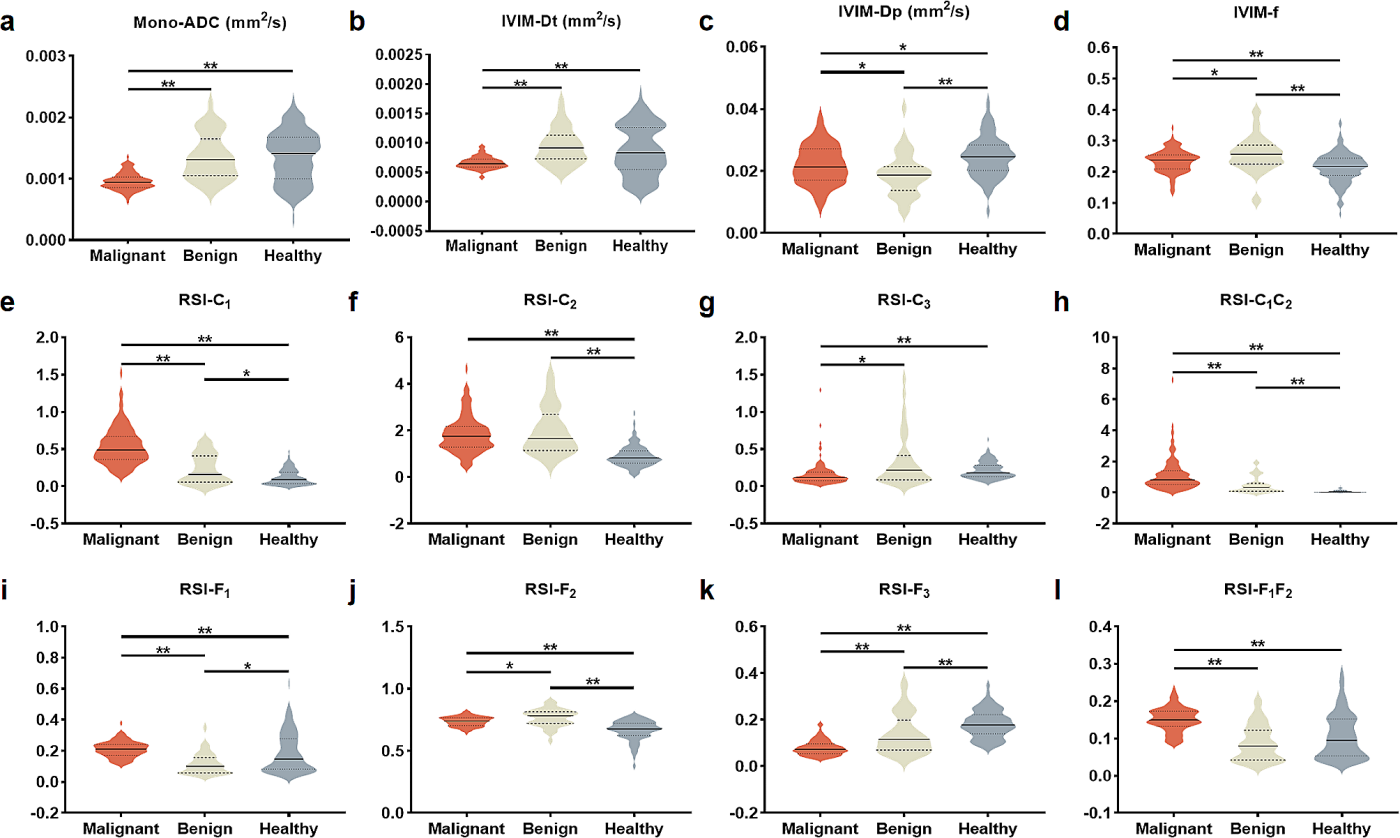
Violin graphs showing the significant quantitative metrics from mono-exponential (**a**), IVIM (**b-d**), and RSI (**e-l**) diffusion models among the malignant tumors, benign lesions, and healthy breast tissues groups. Significance from Mann-Whitney U-test are indicated by the black bars and asterisks (*P < 0.0167, **P < 0.001). ADC, apparent diffusion coefficient; IVIM, intravoxel incoherent motion; RSI, restriction spectrum imaging

The parameter values of the mono-exponential, IVIM, and RSI models in the different groups are presented in Table 4; Fig. 1. The Kruskal–Wallis test indicated that all diffusion parameters derived from the mono-exponential model (ADC), IVIM model (Dt, Dp, and f), and RSI model (C_1_, C_2_, C_3_, C_1_C_2_, F_1_, F_2_, F_3_, and F_1_F_2_) exhibited significant differences among malignant tumors, benign lesions, and normal breast tissues (all *P* < 0.001; Table 4). Upon further comparisons between specific group pairs, it was observed that all quantitative diffusion parameters exhibited significant differences in distinguishing malignant tumors from benign lesions and normal tissues, except for C_2_. Significantly higher C_1_, C_1_C_2_, F_1_, F_1_F_2_ and lower ADC, Dt, C_3_ and F_3_ were found in the malignant tumors, compared to the benign lesions and normal breast tissues (all *P* < 0.005).

**Table 4:** The descriptive statistics of the mono-exponential, IVIM and RSI derived parameters

	Malignant	Benign	Healthy	χ^2^ value	P value*	Z value (P value**)		
						Malignant vs. Benign	Malignant vs. Healthy	Benign vs. Healthy
Mono-ADC (×10^− 3^ mm^2^/s)	0.955 ± 0.140	1.357 ± 0.367	1.362 ± 0.407	81.620	**< 0.001**	-6.714 **(< 0.001)**	-8.267 **(< 0.001)**	-0.219 (0.827)
IVIM-Dt (×10^− 3^ mm^2^/s)	0.662 ± 0.106	0.940 ± 0.304	0.868 ± 0.420	31.310	**< 0.001**	-5.778 **(< 0.001)**	-4.111 **(< 0.001)**	-1.122 (0.262)
IVIM-Dp (mm^2^/s)	0.022 ± 0.006	0.019 ± 0.007	0.025 ± 0.007	27.008	**< 0.001**	-2.717 **(0.007)**	-3.117 **(0.002)**	-4.850 **(< 0.001)**
IVIM-f	0.235 ± 0.036	0.256 ± 0.059	0.213 ± 0.048	31.206	**< 0.001**	-2.546 **(0.011)**	-4.054 **(< 0.001)**	-4.675 **(< 0.001)**
RSI-C_1_	0.540 ± 0.250	0.233 ± 0.195	0.119 ± 0.102	178.453	**< 0.001**	-6.334 **(< 0.001)**	-13.223 **(< 0.001)**	-3.157 **(0.002)**
RSI-C_2_	1.826 ± 0.778	1.972 ± 1.0008	0.894 ± 0.444	127.860	**< 0.001**	-0.248 (0.804)	-10.477 **(< 0.001)**	-7.161 **(< 0.001)**
RSI-C_3_	0.161 ± 0.159	0.329 ± 0.345	0.215 ± 0.110	29.935	**< 0.001**	-2.789 **(0.005)**	-5.494 **(< 0.001)**	-0.044 (0.965)
RSI-C_1_C_2_	1.151 ± 1.042	0.484 ± 0.535	0.078 ± 0.082	195.292	**< 0.001**	-5.083 **(< 0.001)**	-13.611 **(< 0.001)**	-5.878 **(< 0.001)**
RSI-F_1_	0.209 ± 0.051	0.116 ± 0.068	0.184 ± 0.123	41.670	**< 0.001**	-7.045 **(< 0.001)**	-3.602 **(< 0.001)**	-3.117 **(0.002)**
RSI-F_2_	0.737 ± 0.039	0.767 ± 0.069	0.665 ± 0.081	91.203	**< 0.001**	-3.089 **(0.002)**	-8.261 **(< 0.001)**	-6.689 **(< 0.001)**
RSI-F_3_	0.079 ± 0.032	0.138 ± 0.085	0.182 ± 0.059	154.082	**< 0.001**	-4.076 **(< 0.001)**	-12.508 **(< 0.001)**	-3.925 **(< 0.001)**
RSI-F_1_F_2_	0.151 ± 0.032	0.088 ± 0.048	0.106 ± 0.060	67.776	**< 0.001**	-6.678 **(< 0.001)**	-7.081 **(< 0.001)**	-1.697 (0.090)

Note: Data are presented as mean ± SD. *P* values* are calculated by Kruskal-Wallis test, and a *P* value < 0.05 was considered statistically significant. *P* value** are calculated by Mann-Whitney U-test, and a *P* value < 0.0167 was considered statistically significant. Bolded text indicates statistical significance

**Fig. 2:**
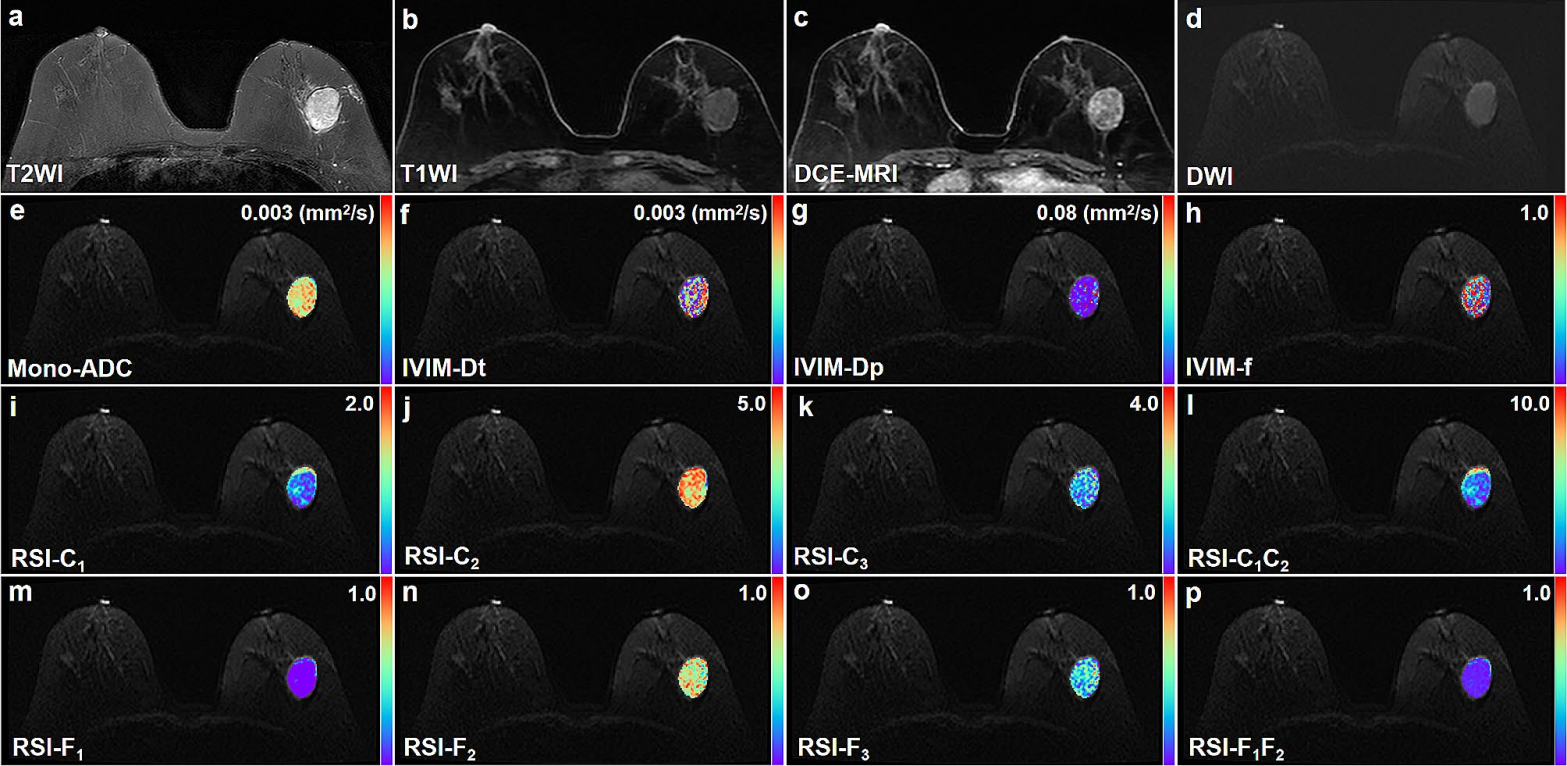
Benign fibroadenoma confirmed by surgical pathology in the right breast of a 56-year-old woman. **a** T2WI. **b** T1WI. **c** DCE-MRI shows an oval mass with heterogeneous enhancement. **d** This mass shows oval shape, smooth margin, and homogeneous internal signal on DWI (b value = 750 s/mm^2^). e-p Pseudo-colorized images shows the Mono-ADC (**e**), IVIM-Dt (**f**), IVIM-Dp (**g**), IVIM-f (**h**), RSI-C_1_(**i**), RSI-C_2_ (**j**), RSI-C_3_ (**k**), RSI-C_1_C_2_ (**l**), RSI-F_1_ (**m**), RSI-F_2_ (**n**), RSI-F_3_ (**o**), and RSI-F_1_F_2_ (**p**) maps derived from mono-exponential model, intravoxel incoherent motion (IVIM), and restriction spectrum imaging (RSI), respectively

**Fig. 3:**
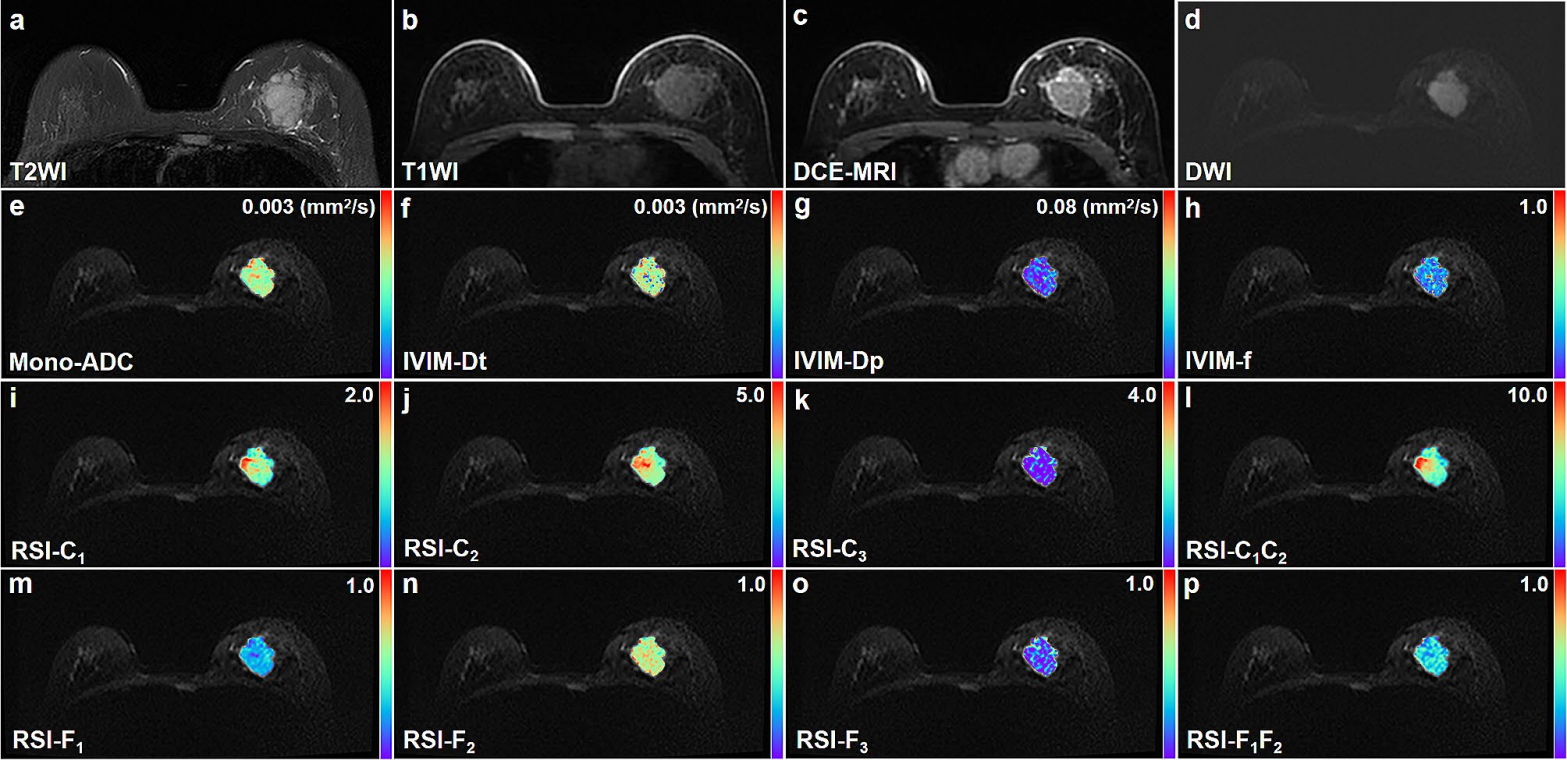
Invasive ductal carcinoma confirmed by surgical pathology in the right breast of a 37-year-old woman. **a** T2WI. **b** T1WI. **c** DCE-MRI shows an irregular mass with obvious enhancement. **d** This mass shows irregular shape, irregular margin, and heterogeneous internal signal on DWI (b value = 750 s/mm^2^). **e-p** Pseudo-colorized images shows the Mono-ADC (**e**), IVIM-Dt (**f**), IVIM-Dp (**g**), IVIM-f (**h**), RSI-C_1_(**i**), RSI-C_2_ (**j**), RSI-C_3_ (**k**), RSI-C_1_C_2_ (**l**), RSI-F_1_ (**m**), RSI-F_2_ (**n**), RSI-F_3_ (**o**), and RSI-F_1_F_2_ (**p**) maps derived from mono-exponential model, intravoxel incoherent motion (IVIM), and restriction spectrum imaging (RSI), respectively

Regarding the comparison between benign lesions and normal tissues, the values of f, C_1_, C_2_, C_1_C_2_, and F_2_ were significantly higher in the benign lesion than in the healthy tissues (all *P* < 0.005). On the other hand, the values of Dp, F_1_, and F_3_ were significantly lower in the benign lesions (all *P* < 0.005). To provide a better understanding, we have included representative images of benign and malignant lesions in Figs. 2 and 3, respectively.

### ROC analysis of the differential diagnostic performance of individual quantitative diffusion parameters and different models

**Fig. 4:**
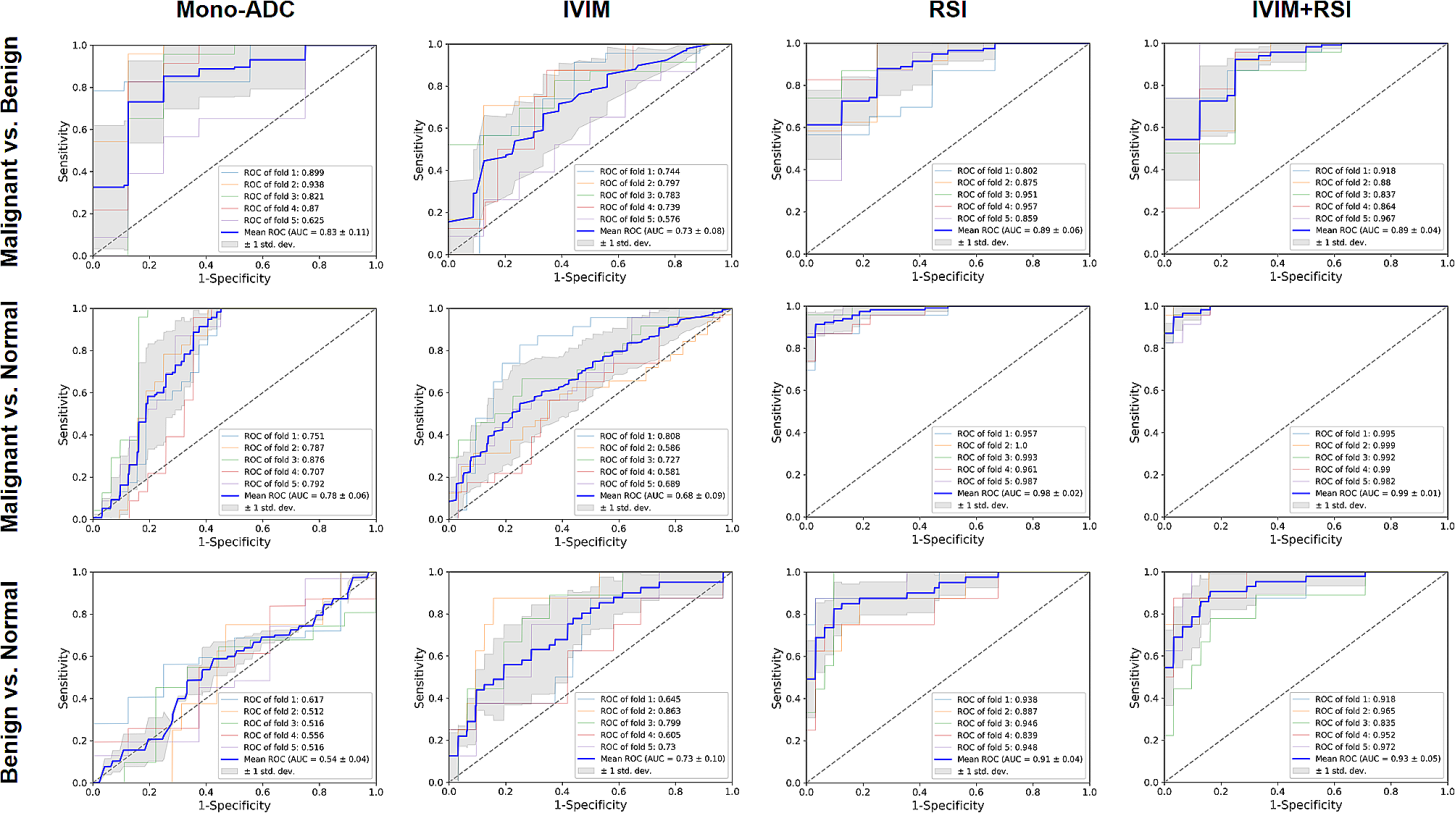
ROC curves and AUC values of different models (mono-exponential model-ADC, IVIM model, RSI model, and IVIM + RSI model) for discriminating malignant and benign lesions, malignant lesions and healthy tissues, benign lesions and healthy tissues on five-fold cross validation. ROC, receiver operating characteristic; AUC, area under the curve; ADC, apparent diffusion coefficient; IVIM, intravoxel incoherent motion; RSI, restriction spectrum imaging

Table 5; Fig. 4 display the results of the ROC analysis conducted on the individual parameters and each model, for the distinguishment of malignant tumors, benign lesions and normal tissues.

**Table 5:** Receiver operating characteristic curve analysis of individual parameters for differential diagnosis

Malignant vs. Benign												
	ADC (×10^− 3^ mm^2^/s)	Dt (×10^− 3^ mm^2^/s)	Dp (mm^2^/s)	f	C_1_	C_2_	C_3_	C_1_C_2_	F_1_	F_2_	F_3_	F_1_F_2_
AUC	0.853	0.804	0.643	0.634	0.833	0.513	0.647	0.767	**0.871**	0.663	0.714	0.851
Threshold	≤ 1.025	≤ 0.729	>0.022	≤ 0.255	>0.343	≤ 2.900	≤ 0.227	>0.503	>0.124	≤ 0.780	≤ 0.106	>0.123
Sensitivity (%)	75.00	78.45	49.14	75.86	80.17	89.66	84.48	75.86	96.55	87.93	81.90	81.03
Specificity (%)	82.93	75.61	78.05	51.22	70.73	24.39	48.78	70.73	65.85	51.22	58.54	80.49
**Malignant vs. Healthy**												
	ADC (×10^− 3^ mm^2^/s)	Dt (×10^− 3^ mm^2^/s)	Dp (mm^2^/s)	f	C_1_	C_2_	C_3_	C_1_C_2_	F_1_	F_2_	F_3_	F_1_F_2_
AUC	0.793	0.646	0.610	0.644	0.968	0.871	0.695	**0.982**	0.628	0.792	0.943	0.751
Threshold	≤ 1.162	≤ 0.791	≤ 0.023	>0.234	>0.281	>1.197	≤ 0.126	>0.330	>0.124	>0.696	≤ 0.129	>0.116
Sensitivity (%)	91.38	90.52	60.34	54.31	88.79	82.76	55.17	87.07	96.55	82.76	93.97	84.48
Specificity (%)	66.88	54.14	59.87	68.79	91.08	79.62	78.34	99.36	44.59	64.33	78.34	61.78
**Benign vs. Healthy**												
	ADC (×10^− 3^ mm^2^/s)	Dt (×10^− 3^ mm^2^/s)	Dp (mm^2^/s)	f	C_1_	C_2_	C_3_	C_1_C_2_	F_1_	F_2_	F_3_	F_1_F_2_
AUC	0.511	0.557	0.746	0.737	0.660	**0.863**	0.502	0.798	0.658	0.840	0.699	0.586
Threshold	≤ 1.388	>0.472	≤ 0.022	>0.225	>0.302	>0.999	>0.100	>0.330	≤ 0.196	>0.699	≤ 0.140	≤ 0.147
Sensitivity (%)	63.41	100	78.05	80.49	39.02	90.24	65.85	51.22	90.24	87.80	65.85	90.24
Specificity (%)	52.23	21.66	65.61	58.60	94.27	66.88	11.46	99.36	38.22	64.97	75.16	27.39

Note: The best AUC of diffusion parameter in Malignant vs. Benign, Malignant vs. Healthy, and Benign vs. Healthy is shown in bold

When using individual parameters, RSI-derived parameters (F_1_, C_1_C_2_, and C_2_ values) showed the highest AUCs for malignant vs. benign lesions (AUC = 0.871, sensitivity = 96.55%, and specificity = 65.85%), malignant lesions vs. normal tissues (AUC = 0.982, sensitivity = 87.07%, and specificity = 99.36%), and benign lesions vs. normal tissues (AUC = 0.863, sensitivity = 90.24%, and specificity = 66.88%), respectively.

In addition, the five-fold CV analysis showed that the RSI model demonstrated significantly higher AUCs of 0.889, 0.980 and 0.911 for the pairwise classifications, compared to either mono-exponential model (AUCs = 0.830, 0.783, and 0.544, respectively) or IVIM model (AUCs = 0.728, 0.678, and 0.728, respectively) alone. Furthermore, the hybrid model (IVIM + RSI) showed the highest diagnostic efficacy for distinguishing malignant vs. benign lesions, malignant vs. normal tissues, and benign vs. normal tissues (AUCs = 0.893, 0.991, and 0.928, respectively).

## Discussion

This study represents a significant step forward in evaluating the effectiveness of two compartmentalized diffusion-weighted models (IVIM and RSI) for the differential diagnosis of breast lesions, as compared to the conventional mono-exponential model. The results demonstrate that diffusion parameters derived from IVIM and RSI models can effectively distinguish between malignant and benign lesions (excluding C_2_), and normal fibroglandular tissues (all parameters). Notably, RSI-derived parameters showed better diagnostic performance than IVIM-derived parameters and mono-exponential ADC in distinguishing the different tissue types (F_1_ for malignant vs. benign, AUC = 0.871; C_1_C_2_ for malignant vs. normal tissue, AUC = 0.982; C_2_ for benign vs. normal tissue, AUC = 0.863). Additionally, the hybrid model, which integrates the IVIM and RSI models, demonstrated improved diagnostic performance in characterizing the three tissue types compared to individual models (AUC = 0.893, 0.991, and 0.928 for malignant vs. benign, malignant vs. normal tissues, and benign vs. normal tissues, respectively). Additionally, the mean DSC of 0.885 and the high ICCs of all parameters derived from the three models indicated that the whole-lesion analysis covering the entire lesion volume would be objective and reliable.

Substantial studies have indicated that ADC value, calculated from mono-exponential model relied on the premise that the diffusion of water molecules complies with a Gaussian distribution, was thought to be associated with tissue cellularity [2, 3, 7]. Attributable to the high capacity of rapid cell proliferation, malignant tumors have dense cellular density and decreased extracellular space that restricted the water molecules’ free diffusion, contributing to significantly lower ADC value than that of benign lesions and normal breast tissues, which was in agreement with our study. Yet, given the effects of blood microcirculation and the complexity of tissue microstructure, ADC value may not be able to accurately assess the diffusion of water moleculars in biological tissue.

The bi-exponential IVIM model enables the water molecular diffusion to separate into two diffusion components (true diffusion component and pseudo-perfusion component) and has capability of providing more accurate information about tissue diffusion. Our study results showed that the Dt values (reflecting the true diffusion of water molecules) were significantly lower than ADC values in all three types of breast tissues, which partially reflects the contribution of the microcirculation perfusion to the ADC values. In addition, it is noticeable that Dt values obtained substantially better diagnostic efficiency than ADC values for the differential diagnosis of benign lesions and malignant tumors in this study (AUC = 0.853 for ADC and 0.804 for Dt, *P* = 0.036). Furthermore, it was found that malignant tumors presented significantly lower Dt values than benign lesions (*P* < 0.001), which was in agreement with the meta-analysis reported by Arian et al. and Baxter et al. [10, 30]. This may be related to the vigorous cell proliferation, increased nuclear-cytoplasmic ratio, and decreased intercellular and intracellular space in malignant lesions, which highly refrains water molecule diffusion. Besides, we found that the AUC of Dt value in distinguishing malignant tumors from benign lesions in our study was higher compared to those reported by Ji et al. and Jin et al. [31, 32]. We hypothesized that this discrepancy might be attributed to factors such as the relatively small number of lesions and the 2D delineation of the region of interest (ROI) in their research.

Dp value described the perfusion-related diffusion of blood microcirculation and was correlated with the capillary length and blood flow velocity. Meng et al. reported that Dp values in the malignant tumors group were significantly higher than in benign ones, which is consistent with ours [12]. This may be related to the abundant capillaries in the stroma of malignant diseases. However, a study conducted by Xu et al. reached the opposite conclusion [33]. In addition, the AUC of Dp value in our study surpassed the results reported by He et al., which may be attributed to differences in the selection of the b-values used for data calculation [11]. Previous studies pointed out that the Dp value could be influenced by the T2 contribution, neighboring tissue structures, and motion artifacts [11, 34]. Therefore, Dp value is not recommended as a reliable imaging indicator for the differential diagnosis of breast lesions.

The signal intensity ratio of microcirculation perfusion and overall tissue diffusion could be quantitatively represented by the f value. Our results found that the f values of malignant lesions were significantly lower than benign lesions (*P* < 0.001), which is consistent with Suo et al. [35]. According to a study reported by Jin et al. and Meng et al., the reduced f value in malignant tumors may be attributed to the decrease of normal angiogenesis and the abundance of tumor capillaries, which had the property of disordered branches and were prone to hyperplasia tortuously and compress attribute to the increased cell density [12, 32]. However, the studies conducted by Liang et al. showed opposite results [13]. This is probably the result of variation in the choice of imaging protocols (e.g., the number and range of b values) across multiple centers.

The RSI model recently developed allows the decomposition of the mixture water molecule’s diffusion signal into three components. C_1_ and F_1_ represent the restricted diffusion compartment, correlated with the cellularity of the tumor. Our data showed that C_1_ and F_1_, as well as C_1_C_2_ and F_1_F_2_ of malignant lesions, was significantly higher compared to benign lesions and normal fibroglandular tissues, which is in line with the findings reported by Besser and Qin et al. [17, 22]. In malignant lesions, higher cellularity and nuclear-cytoplasmic ratio might restrict the diffusion of water molecules and thus enhance the volume fraction of restricted diffusion compartment in the microenvironment [11], which could be extracted sensitively by the restricted diffusion parameters at high b-value. Therefore, the RSI model has the potential value in diagnosing malignant lesions and in providing noninvasive measurements of the lesion microstructure and aggressiveness.

In addition, C_2_ and F_2_ were thought to be associated with the signal from the greater hindered diffusion compartment, primarily from fibroglandular tissue [17]. From our data, C_2_ and F_2_ values were higher in both malignant tumors and benign lesions than in that of normal tissues. There was nonsignificant difference between benign lesions and malignant tumors on C_2_. Previous studies have shown that the T2 relaxation time of benign lesions was significantly higher than that of malignant lesions [36, 37]. Therefore, we hypothesize that generally higher C_2_ and F_2_ signal values of benign lesions may be concerned with the higher T2 value and composition of fibroglandular tissue in some types of benign lesions [38].

Furthermore, C_3_ and F_3_ reflected the free diffusion of water molecules in tissue. Our findings showed that significantly lower C_3_ and F_3_ were found in the malignant tumors compared to the benign lesions and normal breast tissues. This possibly occurs due to the hypercellular tissue density, substantial synthesis of macromolecular substances and increased necrotic substances in malignant lesions, leading to reduced extracellular space for the diffusion of free water proton [39].

The ROC analysis for the differentiation malignant tumors from benign lesions revealed that RSI model (AUC = 0.889) obtained substantially better diagnostic efficacy than mono-exponential model and IVIM model (AUC = 0.830 and 0.728, respectively). Notably, when we combined the IVIM model and RSI model, the hybrid models achieved the best diagnostic efficacy (AUC = 0.893). This may be result from the reason that hybrid model has capability to simultaneously explore the cellularity, vascularity, and microstructure of breast tissue by making the best of the full b-value spectrum information.

Discordantly to the preliminary publications by Besser et al. and Jin et al. [17, 32], there are significant differences in some of the parameters of IVIM model and RSI model between benign lesions and normal breast tissue in our research (IVIM-Dp, f; RSI-C_1_, C_2_, C_1_C_2_, F_1_, F_2_, F_3_; all *P* < 0.005). This may be partly related to the differences in delineation of health control ROIs, pathological types of benign lesions, and diffusion coefficients of each compartment. Significantly, the hybrid models we proposed achieved the optimal diagnostic performance (AUC = 0.928), which provide valuable insights for future research and clinical practice.

The present study had some limitations that need to be addressed. Firstly, although we enrolled a relatively large number of gross lesions, the primary histologic types of malignant and benign lesions were invasive carcinomas of no special type and fibroadenomas. Only several other pathological types were included in this study, which might affect statistical results and partially limit the generalization of our findings. Therefore, future analyses should include a broader range of histological types of lesions in a larger cohort to generate more precise and accurate results. Secondly, the diameter of the lesions in our study was larger than 1 cm to avoid partial volume effects and the lesions with non-mass morphological characteristics were not included attributed primarily to the inaccurate delineation and unreliable parameter measurements. However, with the improvement of the DWI acquisition technique, smaller lesions and non-mass lesions should also be accurately evaluated in future research. Thirdly, it was a prospective study conducted at a single institution with all MR scans performed using a single vendor, which may reduce the persuasiveness of the results. Additionally, the selection of b values in diffusion-weighted imaging, particularly in RSI models, varies across studies, and there is currently no consensus on the optimal range or maximum value. Thus, the model needs to be tested in multicenter studies to further validate our results. Lastly, the DWI acquisition parameters with slice thickness of 5 mm was not aligned with the ideal breast MRI protocols (using 4 mm or less) recommended by the European Society of Breast Radiology, which render the DWI performance results less generalizable.

## Conclusions

In conclusion, the results of this study demonstrate the potential of quantitative parameters derived from the three-compartment Restricted Diffusion Spectrum Imaging (RSI) model as imaging indicators for the differential diagnosis of breast lesions and healthy tissues. The hybrid model that integrates Intravoxel Incoherent Motion (IVIM) and RSI, on the other hand, shows superior diagnostic performance by taking into account the cellularity, vascularity, and microstructure of breast tissues. These findings suggest that the hybrid model can be a game-changer in non-contrast-enhanced breast MR imaging by improving the effectiveness of breast cancer screening and reducing the need for unnecessary biopsies.

## Data Availability

No datasets were generated or analysed during the current study.

## Abbreviations

DWI: Diffusion-weighted imaging
MRI: Magnetic resonance imaging
ADC: Apparent diffusion coefficient
IVIM: Intravoxel incoherent motion
RSI: Restriction spectrum imaging
HIS: Hospital Information System
DCE-MRI: Dynamic contrast-enhanced magnetic resonance imaging
FSE: Fast spin-echo
ss-EPI: Single-shot echo-planar imaging
ROI: Regions of interest
DSC: Dice similarity coefficient
ICC: Intraclass correlation coefficient
CV: Cross-validation
ROC: Receiver operating characteristic curve
AUC: Area under the curves
T2WI: T2-weighted imaging
